# The National Clinical Impact Awards: cosmetic change or fundamental reform?

**DOI:** 10.1177/01410768221108499

**Published:** 2022-07-07

**Authors:** Ryan Essex, Mala Rao, Fraz Mir, Surash Surash, Mark Exworthy

**Affiliations:** 1 Institute for Lifecourse Development, The University of Greenwich, London, SE10 9LS, UK; 2 Director, Ethnicity and Health Unit, Department of Primary Care and Public Health, Imperial College London, London, W6 6RP, UK; 3 Cambridge University Hospitals NHS Foundation Trust, Cambridge, UK; 4 Newcastle Hospitals NHS Foundation Trust, Newcastle, UK; 5 Health Services Management Centre, University of Birmingham, Birmingham, B15 2RT, UK

In January this year, the UK government announced the newly named National Clinical Impact Awards (NCIAs), formerly the Clinical Excellence Awards (CEAs) and until 2003 the Distinction Award Scheme. While this new form of the scheme introduced a number of changes, like previous iterations, it largely remains a pay for performance scheme with consultants and academic general practitioners eligible to apply. Applicants can apply to be awarded at one of several levels, with higher levels contributing substantially to overall salary. The stated aims of the new scheme were to (1) broaden access, (2) make the application process simpler, fairer and more inclusive, and (3) ensure the scheme rewards and incentivises excellence across a broader range of work and behaviours.^1^ See Box 1 for a summary that explains the key features of NCIAs.

Some changes from CEAs include an increase in the number of NCIAs, up to 600 p.a. (up from 300; although there were also approximately 600 CEAs awarded yearly before 2010). NCIAs will not be renewable and holders will need to re-apply after five years. The ‘bronze’ level award was also dropped from the NCIAs. To improve access to the scheme, NCIAs will ‘more closely monitor applications and improve reporting mechanisms’ and pay awards at full value – regardless of whether the award holder works full-time – and improve ‘training’ for assessment panels regarding issues of diversity. There was also a shift in the domains that applicants would be assessed against, namely (1) developing and delivering service, (2) leadership, (3) education, training and people development, (4) innovation and research, and (5) nationally or internationally recognised quality improvement. Finally, the NCIA scheme cuts the link between local and national awards, with local awards now within the remit of NHS Trusts.^2^ See Box 2 for a summary of these changes.

Regardless of the form these awards have taken or the label applied to them, controversy has not been far behind. One of the earliest studies to shed light on the problematic nature of these awards was published in 1980 by Bruggen and Bourne,^3^ which revealed an ‘iniquitous’ allocations of awards, with younger consultants, certain specialties (for example, obstetrics and gynaecology, among others), women and those in non-teaching hospitals all far less likely to receive an award. The study also identified several issues with the award process, noting that these inequities were at least in part compounded by award committees which had little to no diversity. Subsequent studies have reached similar conclusions.^4^ While several changes have been made over the years, many similar problems persist despite these reforms over four decades later. CEAs were widely criticised for, among other things, inequalities among race, gender and between specialties, with female and ethnic minority applicants underrepresented at all award levels. In addition to the CEA outcomes published yearly in the Advisory Committee on Clinical Excellence Awards (ACCEA) annual report, which invariably contain these disparities (e.g. ACCEA^5^), several other recent reports have been critical of the scheme. The 2020 Mend the Gap report, for example, found that the CEAs contributed toward the broader 20% pay gap in medicine between genders.^6^ Anecdotal evidence also suggests that those who work over and above expected standards clinically are less likely to apply compared to academics. Looking at both national and local CEAs, the 2019 Surash Pearce report found that within their Trust for both local and national CEAs, ethnic minority consultants fared the worst in relation to the average annual value of local awards when compared to their white colleagues.^7^ The disparity was 24.5% for local awards, which went down to 5.4% for national awards. Given these disparities, criticism has also been raised about the nature of the application process, namely the dubiety of subjective scoring by panels (giving the appearance of objectivity), committees made up of (in theory) 50% medical professionals (reform in the 2000s increased lay and managerial representation),^8^ the fact that CEAs far exceed other pay for performance schemes (which are generally <5% of an individual’s salary)^9^ and how excellence is measured, among the other domains against which applicants are assessed.^8,10^ A further criticism is that this scheme rewards individuals working in what is an increasingly team-focused environment in today’s NHS.

It was in this context that reform was sought. Will these changes address the new aims of the scheme? While we are yet to see its outcomes, there is reason to be sceptical. While changes such as increasing the number of awards, providing ‘improved guidance materials, alongside a communications strategy to raise greater awareness of the scheme’ and improved reporting of data^11^ are welcome, there are no firm commitments or goals to address longstanding gender and ethnic disparities with the new changes appearing cosmetic. For example, in training assessors and providing greater guidance, it remains unclear what will be reviewed, what this training entails or whether ethnic minority doctors will be consulted in this process. Nothing has been said about mechanisms to address gender and ethnic disparities. Questions also remain about how one could be trained to equitably measure ‘impact’ across a range of specialties. It also seems naïve to believe that these inequities occur because those who are underrepresented are not aware of the scheme; for example, the Surash Pearce report^7^ found that ethnic minority consultants were more likely to apply for CEAs but less likely to receive an award. The severance of NCIAs from local schemes is also a cause for concern. The value and distribution of local awards have long been opaque, this separation is only likely to exacerbate these problems. Again, the Surash Pearce^7^ report found that the value of local awards on average, for white consultants was 24.5% more compared to ethnic minority colleagues. Beyond these concerns, there is notably no discussion about other groups who are particularly marginalised by the scheme, for example female consultants from ethnic minority^7^ backgrounds and international medical graduates.

Like its predecessors, NCIAs have little evidence to support their ongoing use and effectiveness. Research has been commissioned by NIHR to examine ‘our scoring mechanisms’ to ‘ensure that our scoring processes are fair and non-discriminatory, reflect the right balance of breadth and depth of achievement and that the scoring process as used in the current scheme is understandable to both applicants and assessors’. This is of course a step in the right direction; however, it again falls short. The narrow focus of this investigation still overlooks the most critical elements of these awards, namely whether they improve the delivery of care. Furthermore, we are somewhat sceptical that ‘excellence’ or ‘impact’ can be measured in any reliable way. Traditionally, there has been an element of double-counting in domains and often a lack of clarity as to what constitutes ‘over and above’ contractual duties (even for national awards). This adds to existing concerns about subjectivity of scoring. Furthermore, the fact that CEAs were awarded for ‘excellence’ becomes increasingly harder to justify given the fact that >61% consultants received a local or national award.^8^ The change of focus to ‘impact’ may be significant, but it may also be a semantic change; how this is interpreted will be critical. In saying this, it seems an opportunity was missed to more broadly scrutinise whether NCIAs could be evidence based.

In addition to these points, there are historical reasons for scepticism. Regardless of the form these awards have taken or the label applied to them, many pervasive inequalities remain related to gender, ethnicity, specialty,^4,5^ inequities in the application process and how ‘excellence’ is measured, among the other domains against which applicants are assessed.^13^ Despite attempts at reform, these problems have persisted, perhaps because the government and medical profession rely on each other – the former to provide safe, protected space (including NCIAs) for medicine, the latter to deliver an electorally popular health service.^14^ Moreover, the introduction of NCIAs is particularly insensitive to the needs of the wider NHS workforce. More than ever, healthcare is reliant on teamwork and the past two years of the COVID-19 pandemic has shown graphically where additional support (financial and otherwise) could be spent. There is a case for physicians, and particularly those who are relatively senior to use their position to advocate for the broader good and the NHS.^15,16^ To this extent, the NCIA reforms seem tokenistic and represent a missed opportunity to abolish this scheme and usher in a fairer pay scheme for all, one that recognises the collective effort of teams rather than individuals.

## Figures and Tables

**Table:** **Box 1.** What are National Clinical Impact Awards?

The National Clinical Impact Awards (NCIAs) were announced in 2022 after consultation in 2021. The newly announced awards seek to ‘retain skilled, dedicated clinicians who lead in the provision and improvement of patient care through their innovation and partnership across the NHS, the life sciences industries and through patient involvement’.^11^ Like the Clinical Excellence Awards (CEAs) that preceded them, the NCIAs operate in England and Wales. Also similar to its predecessor, the CEAs, the NCIA scheme provides awards at three levels: ‘silver, gold and platinum’. The NCIA proposes to increase the number of awards to 600 a year (up from the current 300) with awards having to be renewed every five years. This means that up to 3000 consultants or 6% of the eligible consultant population could hold an NCIA at any time. For a more comprehensive history about the history of these awards and CEAs see Essex et al.^10^ and Exworthy et al.^8^

**Table:** **Box 2.** A comparison of Clinical Excellence Awards and National Clinical Impact Awards.

**Clinical Excellence Awards**Awarded on 12 levels: eight local and three national (bronze level awarded locally and nationally)Renewal after 4/5 years. In the 2019/2020 rounds, 84% of CEA renewals were re-awarded at the same or different levels^17^ Per annum, 300 awards (post-2010)Value of silver, gold, platinum awards higher. Awards paid on a full-time equivalent basisAssessed on five domains: (1) service delivery, (2) service development, (3) leadership, (4) research and innovation, and (5) teaching and training^13^	**National Clinical Impact Awards**Awarded on 11 levels: bronze awards abolishedRenewal abolished. Re-application after five yearsPer annum, 600 awardsValue of silver, gold and platinum awards reduced. Awards to be paid in full whether or not the holder works full time.Assessed on five domains: (1) developing and delivering service, (2) leadership, (3) education, training and people development, (4) innovation and research, and (5) nationally or internationally recognised quality improvement.To address ethnicity, gender and other gaps, NCIA scheme will ‘more closely monitor applications and improve reporting mechanisms’

## Declarations

### Guarantor

RE.

### Provenance

Not commissioned; editorial review.

### ORCID iD

Ryan Essex https://orcid.org/0000-0003-3497-3137
